# Condom use in penile-vaginal intercourse among Canadian adults: Results from the sex in Canada survey

**DOI:** 10.1371/journal.pone.0228981

**Published:** 2020-02-20

**Authors:** Tina Fetner, Michelle Dion, Melanie Heath, Nicole Andrejek, Sarah L. Newell, Max Stick

**Affiliations:** 1 Sociology, McMaster University, Hamilton, Ontario, Canada; 2 Political Science, McMaster University, Hamilton, Ontario, Canada; 3 Health Sciences, McMaster University, Hamilton, Ontario, Canada; Pontificia Universidade Catolica do Rio Grande do Sul, BRAZIL

## Abstract

**Purpose:**

This paper examines condom use in penile-vaginal sexual intercourse among adults in Canada.

**Data and method:**

The Sex in Canada survey is a national survey of Canadian adults, ages 18+ (N = 2,303). The online survey used quota-based population sample matching of 2016 census targets for gender, age, region, language, visible minority status, and education level. We report general patterns of self-reported condom use, as well as results from zero-inflated negative binomial regression models on the relationship between condom use and social location, relationship status, and sexual health.

**Results:**

Condom use varies by gender, age, education, visible minority status, and relationship status. Use of condoms is related to the perception of risk of being diagnosed with a sexually transmitted infection in the next six months and to the experience of receiving lessons in condom use. No significant associations were found between condom use and region, rural/urban residence, income, or religion. Among men, but not women, condom use is associated with language preference, past diagnosis with a sexually transmitted infection, and self-reported sexual health.

**Conclusion:**

Canadian adults report using a condom in approximately 30% of their sexual encounters involving penile-vaginal sex. Condom use is highest among young adults. Single people use condoms more often than people with marital or common-law partners. Condom use is higher among those with higher levels of education, among people belonging to visible minorities relative to white people, and for men relative to women. People who think they are likely to be diagnosed with a sexually transmitted infection in the next six months are more likely to use condoms than those who do not.

## Introduction

Condoms are a vital component of public health approaches to contraception and the reduction of transmission of sexually transmitted infections (STIs) [1]. Many sexuality education programs tout condom use as a low-cost, hormone-free, and accessible option for pregnancy and STI-transmission prevention [2]. As such, many studies have tracked condom usage among specific populations of interest, as well as the general population [3–5]. Condom usage surveys allow public health officials, sexuality educators, policy makers and the public at large to understand the impact of programmatic interventions in STI and pregnancy prevention, including safer sex education curricula, condom distribution programs, and public awareness campaigns.

Proper and consistent use of condoms were particularly central to public health officials’ response to the human immunodeficiency virus (HIV) epidemic that began in the 1980s, as condoms are uniquely effective at reducing rates of HIV transmission [6]. Rates of condom use, and the conditions under which sexual partners are likely to use condoms, are important social facts for scholars of sexuality, public health experts and policy makers. However, data on condom use in Canada is limited. The Canadian Community Health Survey occasionally includes generalizable information on condom use in Canada [5], but most other studies only address subpopulations such as adolescents [7], midlife adults [8], Indigenous peoples [9,10], or sex workers [11]. Updated data on condom use is helpful to a wide variety of scholars, public health professionals, and publics. This study examines condom use in penile-vaginal sexual intercourse in a representative sample of Canadian adults. Consistent with the literature on this topic, we use the term “condoms” to refer to male condoms, which are worn on the penis. This study does not address the use of “female condoms” which are inserted into the vagina. In addition to providing overall rates of condom use, this paper demonstrates the social organization of condom use by examining differences in demographic subcategories, including gender, age, education, and visible minority status. It also examines the relationships between condom use and several sexual health measures, as well as condom education. Thus, this paper contributes important information for public health in Canada.

### Demographic predictors of condom use

Despite growing concerns about the rise in sexually transmitted infections (STIs), general incidence of condom use is low among adults in the United States and Canada [7,12,13]. In his study of condom use in the United States, Anderson [4] finds that 19% of U.S. adults report using a condom in their last sexual encounter. Reece and colleagues’ [12] more recent National Survey of Sexual Health and Behavior (NSSHB) finds that between 21–25% of adults report using condoms in the most recent sexual encounter, and a 20% rate when asked about their last ten sexual encounters. Researchers in Canada have found similar results on the rates of condom use [13]. Several studies have found that men use condoms more frequently than do women [8,12–14].

Scholars and public health advocates have been concerned with rates of condom use in sexual encounters and the social barriers to their use [12]. For instance, a lack of education regarding condom use may deter people from using condoms [15]. Religious proscriptions against pre-marital sex may interfere with condom use [16]. Socio-demographic forces of various kinds, such as living in a rural area or having low education may negatively impact condom use [17]. Research suggests that reported condom use varies based on gender, with men reporting more condom use than women during their last sexual encounter involving penile-vaginal intercourse [7]. For instance, Canadian studies focused on sexually active 20–34 year olds [7] and university students [13] find that men are significantly more likely than women to report using condoms.

Age cohort is also important to condom use: adolescents are the most likely to use condoms, and older adults are the least [12,18]. In Canada, just under a half (47.2%) of 18–24 year old students report using a condom during their last penile-vaginal sexual encounter [13]. In their study of older cohorts, McKay, Quinn-Nilas and Milhausen [8] find that single, midlife Canadians use condoms at a rate of 35.3% among men and 27.6% among women aged 40–59. Similar to studies in the United States [12], Canadian research shows that condom use declines with age, with a significant drop in condom use among men ages 55–59 [8]. Nonetheless, although the reported rate of condom use among ages 18–24 is higher than in older cohorts, the rate is low across the lifespan of Canadian populations [19]. Research on the relationship between sexual health and condoms in the United States and Canada has paid a great deal of attention to youth, adolescents, and young adults [3,7]. Although researching young people’s condom use is important for understanding the impacts of early intervention, there is a need to understand condom use throughout the life course.

Further, the type of relationship between sexual partners impacts condom use [20]. Individuals are most likely to use a condom in casual sexual encounters that occur outside relationships, compared to marital or similarly committed relationships [8,21]. Those engaging in casual sex were twice as likely to use a condom than those having sex in relationships [4,12]. Similarly, research on university hookups shows that over the course of a university career, students experience a decline in condom use [22]. Women engaging in sexual encounters only with casual partners are the most likely to use condoms, and women with a long-term partner are the least likely [23].

### Sexual health and condom use

Condoms are a highly effective method for preventing the transmission of STIs, including HIV [6,24]. Yet, a recent study of adult Canadian men and women found that perceptions of risk for STIs is low, with 87% responding that they are not very or not at all concerned about contracting a sexually transmitted infection [8]. However, the greater the perceived risk, the more likely it is that an individual will use condoms during sex [25]. For example, event-level research has found that when individuals get tested at sexual health clinics they become more aware of sexual risks, and are therefore more likely to use condoms to avoid their perceived risk of contracting an STI from their partners [25]. On the other hand, Nesoff, Dunkle, and Lang [23] find that being diagnosed with a STI is not consistently associated with condom use.

### Other contraception and condom lessons

Canadian university students report that pregnancy prevention is the most frequently cited reason for using condoms [13]. Hoefnagels and colleagues’ [26] study on self-reported behavioral intentions to use condoms among university students in the Netherlands finds that one third of their respondents who intended to use a condom in a hypothetical encounter with a new sexual partner changed their mind when being told that there was no pregnancy risk involved. What this suggests is that pregnancy may be more impactful than the risk of contracting an STI on condom use decision-making. Similarly, using semi-structured interviews with 13 women aged 18–24, Bolton, McKay and Schneider [27] find that condom use drops when women in dating relationships begin using oral contraceptives. Pregnancy risk among women in dating relationships is a greater concern than STIs, at least in part because there is an assumption of monogamy, which plays an important role in young women’s decision to discontinue using condoms. Research on at-risk young adults in the US finds that less than 20% of their respondents use condoms primarily for disease prevention, one half use condoms primarily for pregnancy prevention, and one third use condoms for both equally [20]. Finally, Nesoff, Dunkle, and Lang [23] also find that hormonal contraceptives decrease condom use. It is therefore relevant to investigate whether being on another form of birth control, particularly oral/hormonal contraceptives, has an impact on condom use.

School-based sex education has been shown to have a positive impact on attitudes towards using condoms, condom usage and sexual health outcomes [28–30]. Individuals who had school-based sex education are more knowledgeable about condom use and are more likely to use condoms during their first sexual encounter than those who received an abstinence-based curriculum [30,31]. Likewise, school-based education on condom use is associated with greater likelihood of getting tested for an STI and less likelihood of being diagnosed with an STI [15]. Further, Weinstein, Walsh and Ward’s [30] study of undergraduate students found an association between women who received sex education and their assertiveness and confidence with condom use, concluding that women who have more knowledge about sexual health are more able to communicate their need for safer sex practices in partnered sex.

The current study offers an update to condom usage data with a new, original survey conducted in 2018. We examine demographic characteristics that reflect social divisions in rates of condom use among adult Canadians. We also examine the effect of self-rated sexual health, perception of risk of being diagnosed with an STI in the near future, and the role of lessons in condom use.

## Methods

Data for this study are from the Sex in Canada research project [32]. This research involving human participants was approved by the McMaster Research Ethics Board, Certificate #2017–113. All participants gave fully informed, written consent to participate. The Sex in Canada survey was administered by Environics Canada, which collaborated with Research Now to deliver the survey to a nationally representative sample of their online panel of 400,000 research participants. Respondents recorded survey responses through an online survey tool, which allowed for anonymity and maximum privacy. The survey questionnaire instrument was a modified version of the questionnaire used by the U.S.-based National Survey of Sexual Health and Behavior [12].

Sampling procedures involved inviting large numbers of panelists to take this survey and filtering volunteers with a series of demographic screening questions that allowed survey takers to fill demographic quotas to produce a sample with proportional similarity to the adult population of Canada at its most recent (2016) census. Our sampling requirements included census matching for gender, visible minority status, age, region of residence, preferred language (English or French), and level of education. In response to emails or notices on the online platform, 6,685 potential respondents initiated the survey, and of those survey starts, 3,317 (49.6%) were deemed to be ineligible due to a filled quota for their demographic group, including representative quotas by province for age, sex, education and visible minority status. Of the 3,368 eligible survey starts, 1,065 (31.6%) abandoned or terminated the survey before completion, while 2,303 (68.4%) respondents completed the entire survey. Of these total respondents, 302 were part of an oversample strata of respondents who self-identified as gay, lesbian, or bisexual, and the remaining 2,001 self-identified as heterosexual or straight. A probability weight is included in all multivariate analyses. While the survey sample size is 2,303, the present study is concerned with condom usage during the last 10 instances of penile-vaginal intercourse during the last year, and 1,187 participants were not asked the question corresponding to our dependent variable for several reasons: they had not engaged in that kind of sex before (362 participants or 15.7% of the total sample), they chose not to answer the question about the type of sex they had engaged in recently (201 participants or 8.7% of the total sample), or they had not engaged in penile-vaginal sexual activity in the past twelve months (502 participants or 21.8% of the total sample. The sample size for the dependent variable after these controlled skips is 1,116 participants. Of those asked about using a condom during their 10 penile-vaginal intercourse experience, 101 participants (4.4% of the total sample) had not had this type of intercourse 10 times, 4 responded “prefer not to answer”, and 15 respondents indicated in response to another question that they had never used a condom before. All these participants were counted as missing, bringing our maximum possible sample size to 1,011, or 43.9% of our full sample.

### Dependent variable

The dependent variable is self-reported condom use during the participant’s ten most recent penile-vaginal sexual intercourse events. Previous research regarding condom use in the United States has used the same dependent variable to examine trends across various groups [12]. In the present survey, participants who engaged in this kind of sexual behaviour in the past twelve months were asked, “Of the last 10 times that you had penile-vaginal intercourse, how many of those times did you use a condom?” with response options including “0 out of 10 times” through “10 out of 10 times,” as well as “I have not done this 10 times” and “prefer not to answer.” Other studies have found that asking about condom use over the past 10 penile-vaginal sexual encounters helps to decrease the potential error that self-reports can produce, compared to the more common measure of condom use at the last sexual encounter [12].

### Independent variables

We examine several demographic and behavioral correlates to condom usage [12].

#### Gender and sexual identity

To record gender identity, we asked respondents the question “What is your current gender identity?” with the responses (1) “Male,” (2) “Female,” (3) “Trans male/Trans man,” (4) “Trans female/ Trans woman” (5) “Genderqueer/Gender non-conforming,” and (6) “Different identity (please state).” After hand recoding the open-ended responses into one of the previous 5 categories, we renamed the first and second responses and combined responses 3 to 5. As a result, gender identity is grouped into the following categories: cisgender men, cisgender women, and trans/gender non-conforming.

As this study measures condom use in penile-vaginal intercourse, same-sex sexual activity is not addressed. Nonetheless, because sexual orientation is an important marker of social location, we control for it in our models. To ascertain sexual orientation we used the question “Which of following commonly used terms best describes your sexual orientation?” with options including (1) “Straight/heterosexual (not gay),” (2) “Gay, lesbian or homosexual,” (3) “Bisexual,” (4) “Asexual (I am not sexually attracted to others),” and (5) “Other, please describe.” We combined “Gay, lesbian or homosexual” and “Bisexual” responses to create a gay/lesbian/bisexual group, and we hand coded open-ended responses into one of the other categories whenever possible. We then created a new variable that combined responses to these two questions, producing five categories: “Heterosexual cisgender women,” “Heterosexual cisgender men,” “Lesbian/bisexual cisgender women,” Gay/bisexual cisgender men,” and “Trans/gender nonconforming of all sexual orientations.” Fifteen respondents who identified as asexual were coded as missing.

#### Age group

To measure age, we asked respondents “What is your year of birth?” Based on their answers, we clustered respondents into the following age groups: 18–24, 25–34, 35–44, 45–54, 55–64, and 65+.

#### Education

Participants were asked “What is the highest level of education that you have attained?” Original response categories were (1) “Less than high school,” (2) “High school diploma,” (3) “Trades or apprenticeship certificate,” (4) “A college or CEGEP degree,” (5) “A university (bachelor’s) degree,” (6) “More than a university degree,” and (7) “Another post-secondary certificate or degree.” We combined these into four categories “High school or less,” “College, trade or technical,” “A university (bachelor’s) degree,” and “More than a university degree.”

#### Visible minority

The Employment Equity Act (S.C. 1995, c. 44) defines the term “visible minority” as “persons, other than Aboriginal peoples, who are non-Caucasian in race or non-white in colour.” To capture racial and ethnic categories we asked respondents “Are you a visible minority?” with the following response options: (1) “No, I am not,” (2) “Yes, I am,” (3) “I am not sure.” We collapsed the first and third response categories, creating a binary variable for “visible minority.”

#### Language preference

At the outset of the survey we asked participants “What language would you like to conduct the survey in? / En quelle langue voulez-vous repondre au sondage?” We used the language choice of participants for the survey as a dichotomous variable for English or French language preference.

#### Relationship status

We asked, “Which of the following best describes your current relationship status?” Respondents were asked to select from (1) “Single and not dating,” (2) “Single and dating/hanging out with someone,” (3) “In a relationship but not living together,” (4) “Living together but not married,” (5) “Married and living together,” (6) “Married but not living together,” and (7) “Other (specify).” We manually reviewed the open responses and sorted them into the most appropriate category. For our analysis we combined responses 4 through 6 as “married or living with a partner.”

#### Sexual health

For sexual health, our survey question stated: “For the purposes of this survey, sexual health refers to physical, emotional, and mental well-being related to sexuality. Overall, would you say your sexual health is…” Responses categories included (1) “Excellent,” (2) “Very Good,” (3) “Good,” (4) “Fair,” and (5) “Poor.”

#### Past STI diagnosis

To account for respondents’ history with STIs we asked, “Have you ever been told by a health care provider that you have/had a sexually transmitted infection (STI) or sexually transmitted disease (STD), such as gonorrhea, chlamydia, or genital herpes?” We collapsed those who answered “No” and “Don’t know” while leaving the “Yes” response category unchanged to create a binary measure.

#### STI in next 6 months

To gauge respondents’ perceptions of risk of contracting an STI, we asked, “What do you think the chances are that you will get a sexually transmitted infection (STI) in the next 6 months?” Responses included (1) “No chance at all,” (2) “Some chance,” (3) “About 50–50,” and (4) “High Chance.” We collapsed the third and fourth category as “50% or greater chance.”

#### Other contraception

We asked respondents, “During the past six months, when you were having penile-vaginal intercourse, which of the following types of protection (contraception) have you or your partner used?” with the option of selecting from a list of contraceptive methods, including “Other.” We then created a binary variable to represent those who used other forms of contraception and those who did not.

#### Condom lessons

We asked respondents, “Have you ever learned how to use a condom or other barrier method from any of the following options:” with possible responses including (1) “Sex education class or health class in high school,” (2) “Sex education class or health class in college or university,” (3) “Physician or other health care provider,” (4) “Book or magazine,” (5) “An adult video (pornography),” (6) “Instructions on or in a condom box or wrapper,” (7) “Instructions included with a “safer sex kit,” (8) “A friend,” (9) “A sex partner,” (10) “A parent or guardian,” (11) “A brother or sister,” (12) “Some other family members,” (13) “The internet,” and (14) “None. I have not learned about condom usage.” We grouped responses into three categories. The first category received informal lessons, including lessons from a book or magazine, an adult video, instructions on a condom box or wrapper, instructions included with a “safer sex kit,” a friend, a sex partner, a parent or guardian, a brother or sister, some other family members, and the internet. The second category received formal lessons, including sex education or health class in high school, sex education class or health class in college or university, and physician or health care provider. The third category included those not receiving lessons, either informal or formal.

#### Region

To determine what region that respondents live in we asked, “What are the first three characters of your postal code?” Using this information, we located the province respondents live in. We then categorized the provinces regionally into: British Columbia, the Prairies (Alberta, Manitoba, Saskatchewan), Ontario, Quebec, and the Atlantic provinces (New Brunswick, Newfoundland, Nova Scotia, and Prince Edward Island).

#### Urban/Rural

A dichotomous variable was also used to distinguish between urban and rural residential location. Respondents reported their Forward Sortation Areas (the first three digits of their postal code). The numeric digit and second letter in this code combine to designate urban or rural residence according to Canada Post.

#### Income

We measured income categorically. In the survey we asked, “What is your best estimate of your total household income from all sources, before taxes and deductions, during the year ending December 31, 2017?” allowing them to select either (1) “Under $20,000,” (2) “$20,001 - $40,000,” (3) “$40,001 - $60,000,” (4) “$60,001 - $80,000,” (5) “$80,001 - $100,000,” or (6) “over $100,001 per year.”

#### Religion

We asked respondents “What is your religion, if any?” offering 14 categories that were grouped into Catholic, Protestant, No religion, and Other/Prefer not to answer.

#### Religious attendance

We measured religious attendance by asking “How often do you attend religious services?” Respondents who claimed a religious identity could choose from (1) “More than once a week,” (2) “Once a week,” (3) “Once or twice a month,” (4) “A few times a year,” (5) “Once a year or less,” or (6) “Never.” Those who did not identify as religious were coded as zero.

### Statistical methods

Because the outcome of interest is not normally distributed and includes a large number of zero responses, we estimate multivariate zero-inflated Poisson regression models of condom use. Zero-inflated models provide multivariate regression coefficient estimates for both the probability that a participant reported zero and the count of condom usage. Probability sampling weights are included in multivariate analyses.

## Results

**Fig 1:**
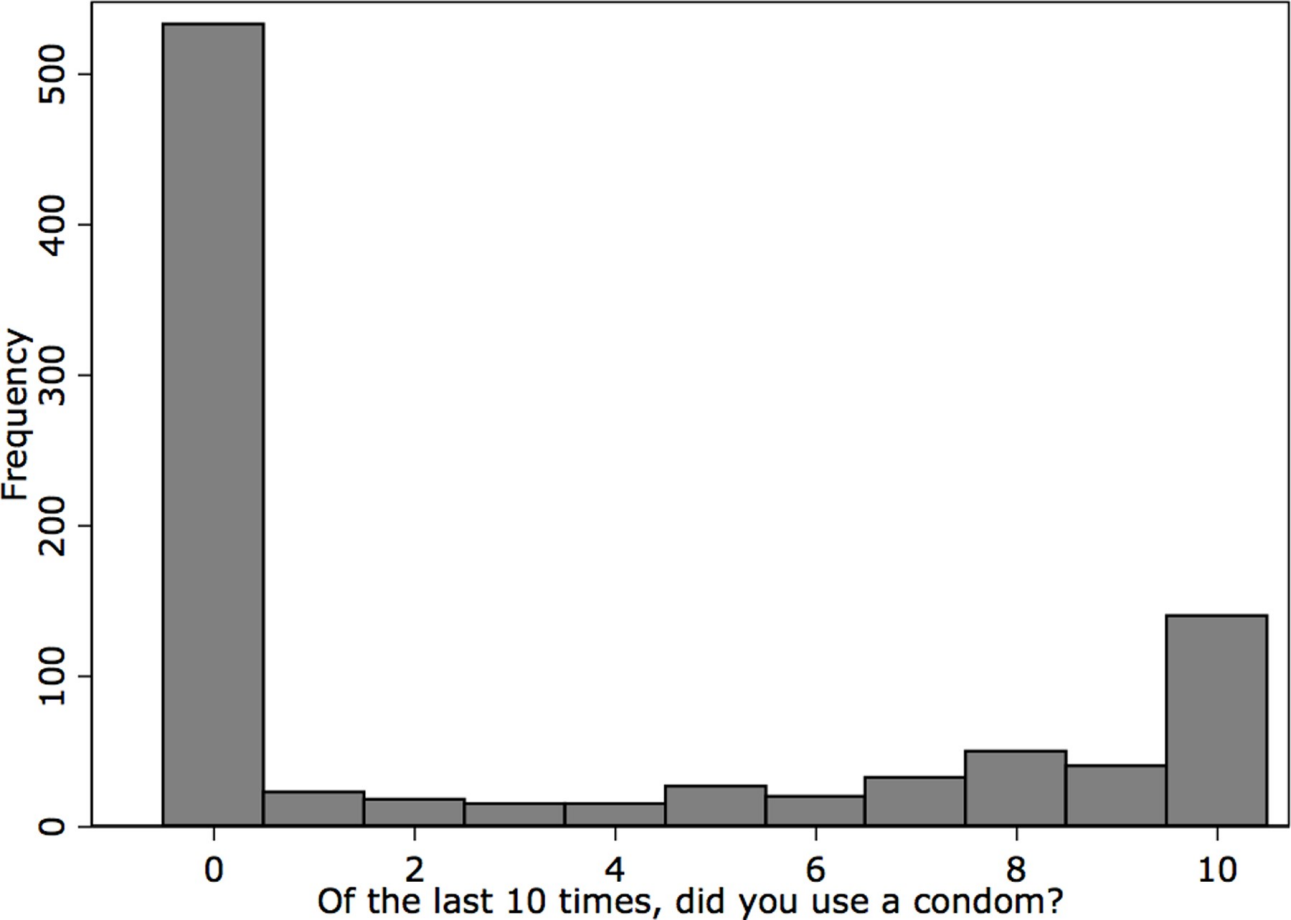
Histogram of condom use, estimation sample.

**Fig 2:**
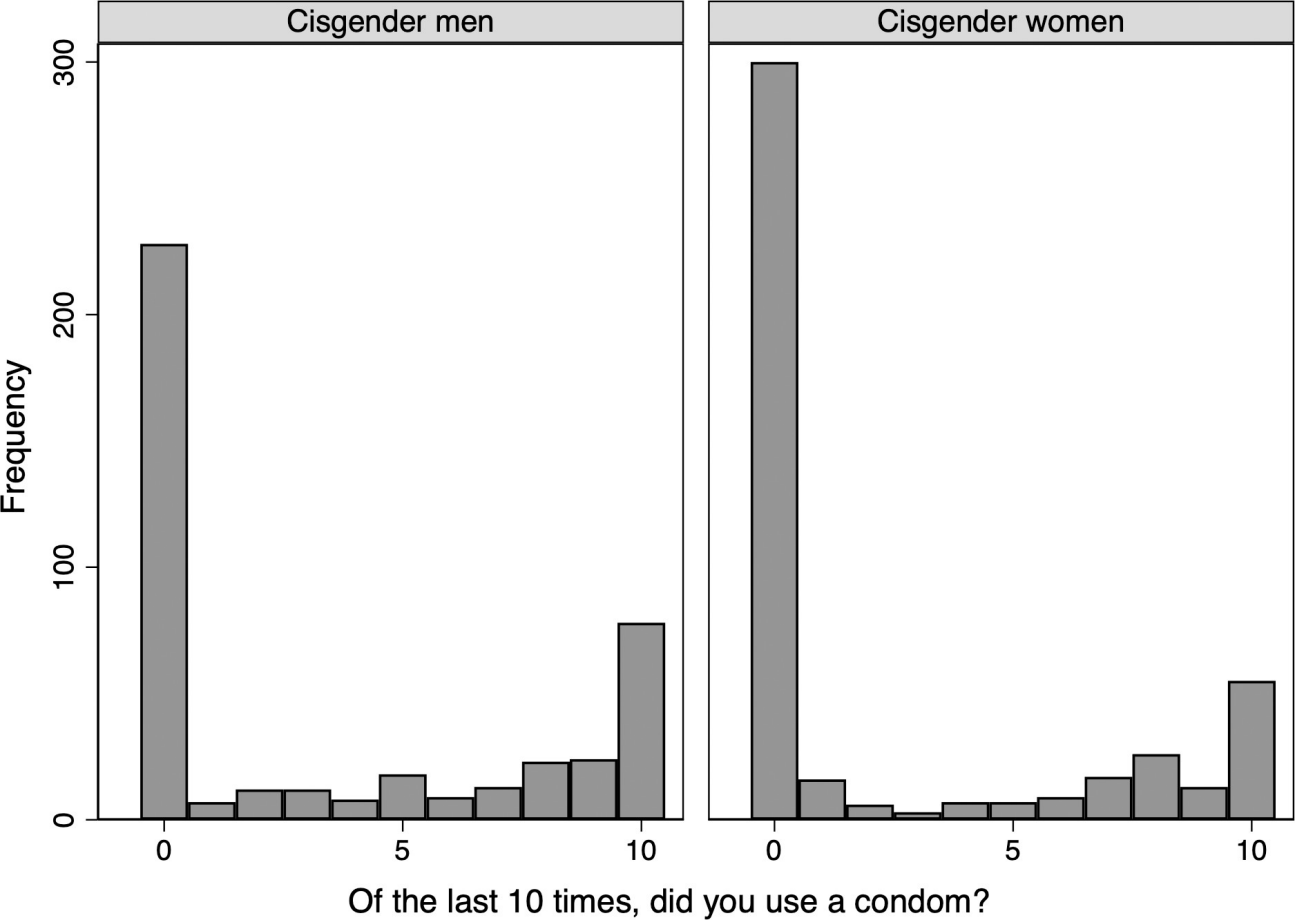
Histogram of condom use, estimation sample by gender.

**Fig 3:**
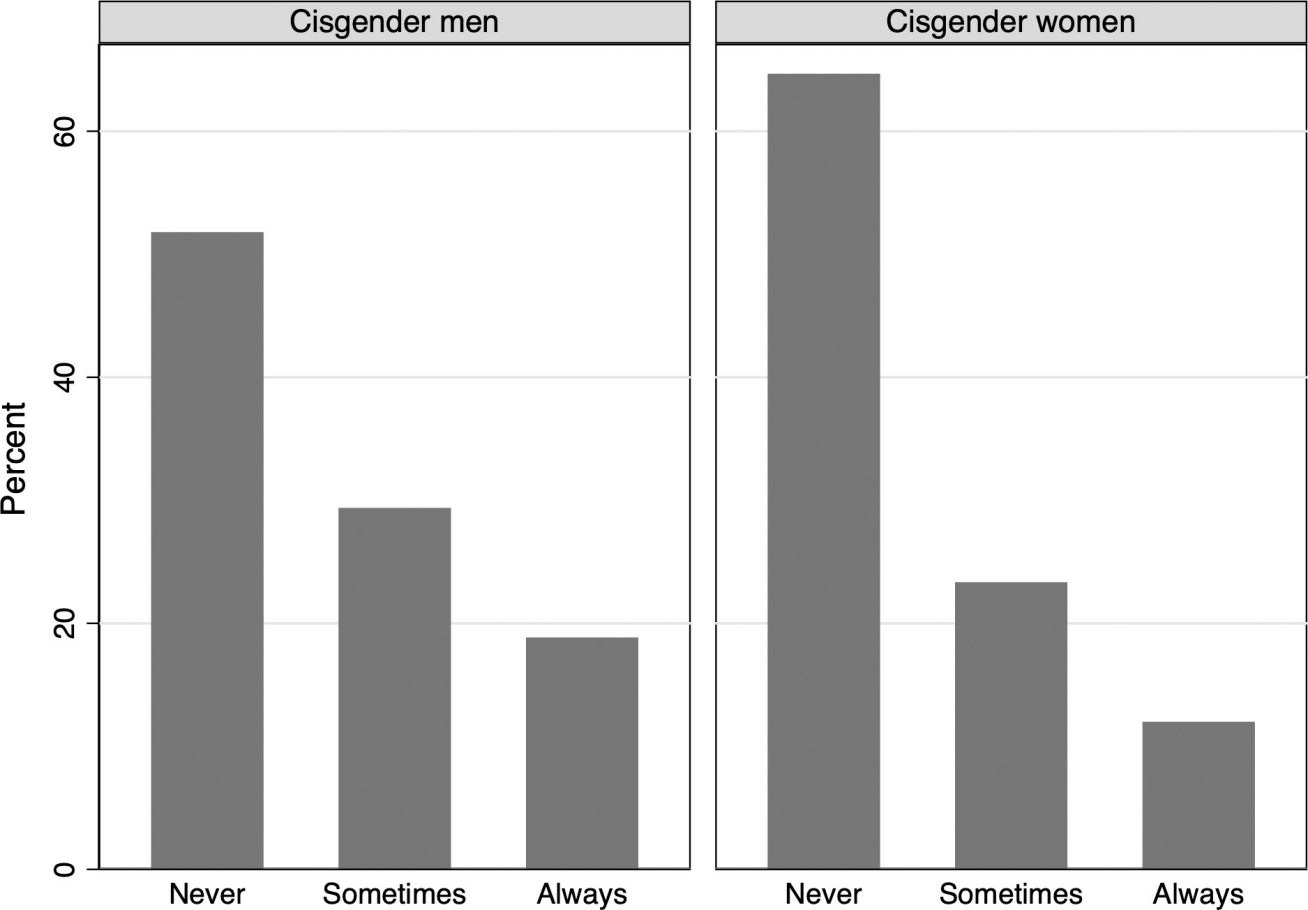
Histogram of ordinal condom use, estimation sample by gender.

**Fig 4:**
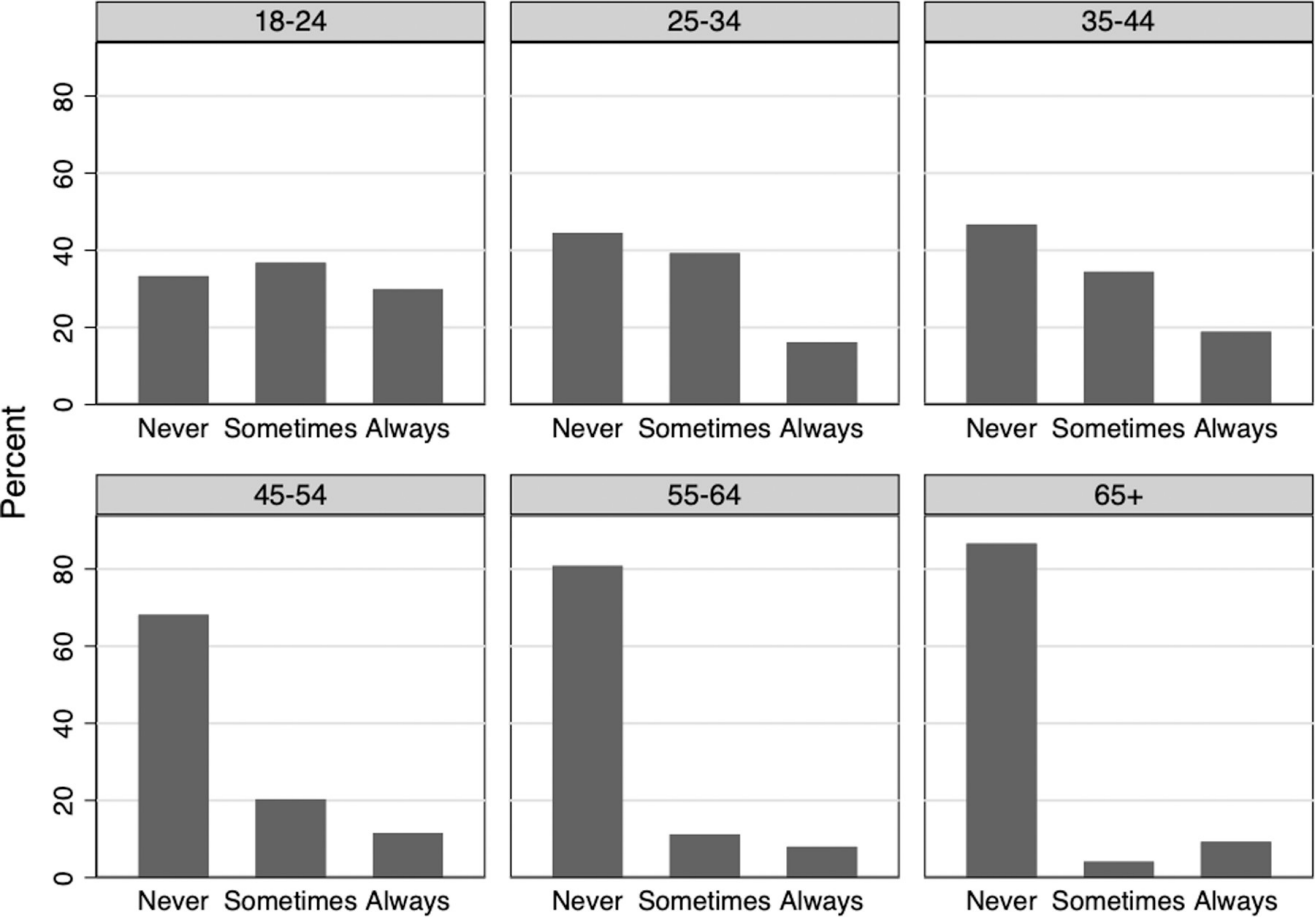
Histogram of ordinal condom use, estimation sample by age.

**Fig 5:**
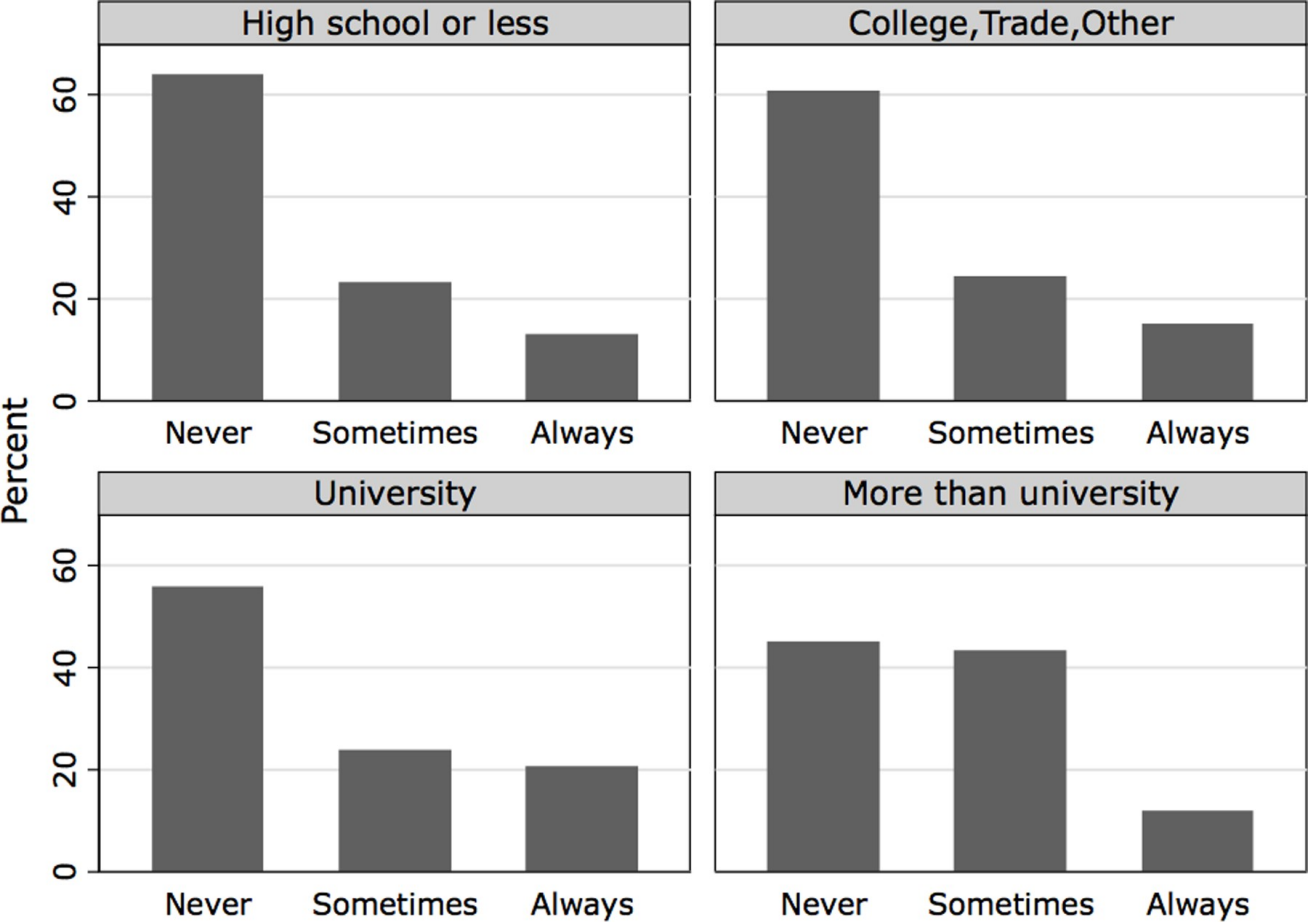
Histogram of ordinal condom use, estimation sample by education.

Of the 1,116 participants who reported engaging in penile-vaginal intercourse within the last twelve months, 4 participants selected prefer not to answer, and 86 participants had not engaged in penile-vaginal sex 10 times (3.9% of the total survey participants), limiting the sample in this analysis to 1,026 participants. The Shapiro Wilk test confirmed that the dependent variable was not normally distributed (restricted to the estimation sample, Z = 8.047, p = 0.000), with a histogram (Fig 1) depicting a large number of respondents reporting zero condom use. The histogram is similar when separating the sample into cisgender men and women respondents, as seen in Fig 2 (While transgender respondents are included in our models, there are insufficient numbers to provide a separate analysis for this group.). To facilitate comparisons of the distribution of condom use across key demographic characteristics, we recode our indicator of condom use as an ordinal measure, with those who responded zero condoms used in the last ten penile-vaginal intercourse experiences as “never,” those who respond 1–9 as “sometimes,” and those who report using condoms ten of ten times as “always.” The distribution is still zero-inflated but to a lesser degree, as seen in Fig 3. When examining ordinal condom use by age, the ‘never’ category becomes increasingly zero-inflated by age category (Fig 4). Ordinal condom use also varies based on education level with the response of never decreasing as education increases (Fig 5).

### Descriptive statistics

Table 1 presents the proportions of dependent and independent variables for the estimation sample, including the sample split into cisgender men and women. Over the past 10 times engaging in penile-vaginal intercourse, participants used condoms on average 3 times, with men having higher rates of use (3.5) than women (2.5) on average. Heterosexual cisgender women represented 44.1% of the sample, heterosexual cisgender men represented 45.2% of the sample, gay or bisexual cisgender men represented 2.4% of the sample, lesbian or bisexual cisgender women represented 6.4% of the sample, with all transgender and gender non-conforming participants representing 1.9% of the sample.

**Table 1:** Descriptive statistics for estimation sample.

	Total	Cisgender male	Cisgender female
	(N = 918)	(N = 437)	(N = 464)
**Of the last 10 times, did you use a condom?**			
Mean (SD)	3.04 (4.07)	3.49 (4.19)	2.51 (3.87)
Min, Max	0, 10	0, 10	0, 10
**Gender & orientation**			
Heterosexual cisgender men	415 (45.2%)	415 (95.0%)	
Gay/bi/pansexual cisgender men	22 (2.4%)	22 (5.0%)	
Heterosexual cisgender women	405 (44.1%)		405 (87.3%)
Lesbian/bi/pansexual cisgender women	59 (6.4%)		59 (12.7%)
Non-conforming/trans all orientations	17 (1.9%)		
**Age**			
18–24	87 (9.5%)	28 (6.4%)	53 (11.4%)
25–34	247 (26.9%)	102 (23.3%)	140 (30.2%)
35–44	180 (19.6%)	94 (21.5%)	84 (18.1%)
45–54	182 (19.8%)	88 (20.1%)	91 (19.6%)
55–64	125 (13.6%)	66 (15.1%)	58 (12.5%)
65+	97 (10.6%)	59 (13.5%)	38 (8.2%)
**Level of Education**			
High school or less	239 (26.0%)	106 (24.3%)	130 (28.0%)
College, trade, or other	360 (39.2%)	173 (39.6%)	180 (38.8%)
University	200 (21.8%)	96 (22.0%)	99 (21.3%)
More than university	119 (13.0%)	62 (14.2%)	55 (11.9%)
**Visible Minority**			
No or Not sure	747 (81.4%)	348 (79.6%)	392 (84.5%)
Yes	171 (18.6%)	89 (20.4%)	72 (15.5%)
**Language**			
French	221 (24.1%)	102 (23.3%)	114 (24.6%)
English	697 (75.9%)	335 (76.7%)	350 (75.4%)
**Relationship**			
Single (not dating/not specified)	99 (10.8%)	57 (13.0%)	38 (8.2%)
Single (dating/hanging out)	96 (10.5%)	48 (11.0%)	43 (9.3%)
In relationship, not living together	92 (10.0%)	35 (8.0%)	54 (11.6%)
Married or living w/partner	631 (68.7%)	297 (68.0%)	329 (70.9%)
**Your sexual health is**			
Poor	31 (3.4%)	11 (2.5%)	20 (4.3%)
Fair	85 (9.3%)	33 (7.6%)	52 (11.2%)
Good	245 (26.7%)	102 (23.3%)	138 (29.7%)
Very good	364 (39.7%)	196 (44.9%)	162 (34.9%)
Excellent	193 (21.0%)	95 (21.7%)	92 (19.8%)
**Ever had STI/STD**			
No/DK	789 (85.9%)	377 (86.3%)	401 (86.4%)
Yes	129 (14.1%)	60 (13.7%)	63 (13.6%)
**Chances of getting STI in next 6 months**			
No chance	751 (81.8%)	342 (78.3%)	401 (86.4%)
Some chance	125 (13.6%)	66 (15.1%)	53 (11.4%)
More than 50%	42 (4.6%)	29 (6.6%)	10 (2.2%)
**Used other contraception**			
No/Unsure/Used condom	764 (83.2%)	376 (86.0%)	376 (81.0%)
Used other contraception	154 (16.8%)	61 (14.0%)	88 (19.0%)
**Condom lessons**			
No lessons	123 (13.4%)	68 (15.6%)	54 (11.6%)
Informal lessons	347 (37.8%)	187 (42.8%)	152 (32.8%)
Formal lessons	448 (48.8%)	182 (41.6%)	258 (55.6%)
**Region**			
BC	107 (11.7%)	44 (10.1%)	61 (13.1%)
Prairies	153 (16.7%)	72 (16.5%)	79 (17.0%)
Ontario	354 (38.6%)	180 (41.2%)	167 (36.0%)
Quebec	252 (27.5%)	119 (27.2%)	129 (27.8%)
Atlantic	52 (5.7%)	22 (5.0%)	28 (6.0%)
**Urban**			
Rural	107 (11.7%)	46 (10.5%)	60 (12.9%)
Urban	811 (88.3%)	391 (89.5%)	404 (87.1%)
**HH income**			
Under $20,000	71 (7.7%)	27 (6.2%)	42 (9.1%)
$20,001-$40,000	129 (14.1%)	54 (12.4%)	75 (16.2%)
$40,001-$60,000	156 (17.0%)	69 (15.8%)	83 (17.9%)
$60,001-$80,000	170 (18.5%)	81 (18.5%)	86 (18.5%)
$80,001-$100,000	168 (18.3%)	87 (19.9%)	77 (16.6%)
Over $100,001	224 (24.4%)	119 (27.2%)	101 (21.8%)
**Religion**			
Catholic	328 (35.7%)	146 (33.4%)	175 (37.7%)
Protestant incl. evan.	161 (17.5%)	83 (19.0%)	73 (15.7%)
Other & PNA	142 (15.5%)	66 (15.1%)	73 (15.7%)
None, Agnostic	287 (31.3%)	142 (32.5%)	143 (30.8%)
**How often do you attend religious services**			
Mean (SD)	1.90 (1.89)	2.09 (1.99)	1.69 (1.76)
Min, Max	0, 6	0, 6	0, 6

The largest age group was those between 18–24 years old (26.9%). 76% of the sample responded in English, and 24% in French. 19% identified as a visible minority. When it came to relationship status, the largest proportion were in a relationship and living together, followed by single and not dating. More than half of the participants live in Ontario or Quebec, with 88% of the sample living in urban areas. The largest group for household income was over $100,000 (24%) with the smallest group being under $20,000 (8%). More than half had a trade certificate, college diploma, or university degree. Catholic respondents represented 36% of the sample and 31% did not identify with any religion. Most participants (82%) believed they have no risk of contracting a sexually transmitted infection in the next 6 months. About 14% had been diagnosed by a medical professional as having a sexually transmitted infection in the past. Almost half (49%) had received formal condom lessons. A large majority (87%) rated their sexual health as good, very good, or excellent.

### Multivariate zero-inflated Poisson regression models

Table 2 includes multivariate regression coefficient estimates for the full sample, cisgender men, and cisgender women for zero-inflated Poisson regression models, including both the logistic regression estimates for an outcome of zero (first column) and the Poisson regression estimates for the condom use count (second column). Positive (negative) coefficients for the logit indicate a characteristic is associated with a higher probability of reporting *not* using a condom (using a condom), whereas positive (negative) Poisson coefficients are associated with *higher* (lower) counts of condom use. For the full sample, the AIC = 2855.19 and BIC = 3260.25. Diagnostic statistics (fitstat, Long and Freese 2014) indicate that the zero-inflated Poisson regression is more appropriate than Poisson, negative binomial or zero-inflated negative binomial for these data.

**Table 2:** Multivariate zero-inflated Poisson regression coefficient estimates for frequency of condom-use in Canada 2018.

Sample	All	All	Cisgender	Men	Cisgender	Women
Estimation	Logit	Poisson	Logit	Poisson	Logit	Poisson
Outcome	Zero	Count	Zero	Count	Zero	Count
**Gender & sexual identity**–Reference: Heterosexual cisgender women (all) & Heterosexual for cisgender samples)						
Heterosexual cisgender men	-0.402*	0.035				
	(0.199)	(0.053)				
Gay/bi/pansexual cisgender men	-1.778	0.208*	-0.948	0.211*		
	(1.050)	(0.094)	(1.416)	(0.087)		
Lesbian/bi/pansexual cisgender women	0.321	0.236***			0.198	0.304***
	(0.540)	(0.066)			(0.472)	(0.082)
Non-conforming/trans all orientations	-0.619	0.113				
	(0.886)	(0.089)				
**Age–**Reference: 18–24						
25–34	0.332	-0.131	1.191	-0.106	0.409	-0.159
	(0.438)	(0.073)	(1.482)	(0.122)	(0.476)	(0.118)
35–44	0.421	-0.117	0.947	-0.116	0.393	-0.056
	(0.456)	(0.074)	(1.538)	(0.125)	(0.515)	(0.101)
45–54	1.510***	-0.105	2.591	-0.160	1.480**	-0.025
	(0.455)	(0.079)	(1.552)	(0.126)	(0.533)	(0.128)
55–64	2.269***	-0.058	2.972	-0.101	3.058***	-0.245
	(0.502)	(0.102)	(1.586)	(0.147)	(0.708)	(0.254)
65+	2.734***	0.044	4.528**	0.172	2.462***	-0.005
	(0.517)	(0.127)	(1.582)	(0.158)	(0.700)	(0.186)
**Highest education**–Reference: High school						
College, trade, or other	-0.572*	0.092	-0.361	0.096	-0.482	0.184
	(0.261)	(0.064)	(0.471)	(0.100)	(0.359)	(0.112)
University	-0.647*	0.153*	-0.576	0.171	-0.645	0.251*
	(0.305)	(0.069)	(0.549)	(0.114)	(0.428)	(0.123)
More than university	-1.201***	0.067	-1.363*	0.129	-1.210*	0.081
	(0.328)	(0.078)	(0.595)	(0.113)	(0.483)	(0.137)
Visible Minority	-0.524*	0.085	-0.417	0.094	-0.527	0.099
	(0.258)	(0.045)	(0.471)	(0.062)	(0.385)	(0.078)
English	0.563	0.086	1.731	0.295*	-0.147	-0.150
	(0.665)	(0.102)	(1.490)	(0.117)	(0.713)	(0.133)
**Relationship status**–Reference Married or living together in a relationship						
Single (not dating/not specified)	-1.969***	0.157**	-3.382***	0.149	-1.441**	0.099
	(0.380)	(0.059)	(0.594)	(0.079)	(0.518)	(0.112)
Single (dating/hanging out)	-1.543***	0.121*	-2.781***	0.018	-1.260*	0.225*
	(0.362)	(0.061)	(0.666)	(0.094)	(0.501)	(0.097)
In relationship, not living together	-0.710*	0.114	-0.714	0.014	-0.660	0.159
	(0.334)	(0.070)	(0.561)	(0.108)	(0.438)	(0.100)
**Sexual health**–Reference: Poor sexual health						
Fair	-1.214	-0.219	-2.217	-0.115	-1.056	-0.313
	(0.649)	(0.215)	(1.357)	(0.181)	(0.787)	(0.341)
Good	-1.279*	-0.087	-2.363*	-0.054	-1.144	-0.058
	(0.597)	(0.203)	(1.054)	(0.148)	(0.720)	(0.330)
Very good	-1.260*	-0.166	-3.002**	-0.060	-0.644	-0.225
	(0.590)	(0.200)	(1.078)	(0.134)	(0.717)	(0.324)
Excellent	-1.152	-0.120	-2.547*	-0.082	-0.648	-0.097
	(0.611)	(0.203)	(1.034)	(0.139)	(0.754)	(0.333)
Ever had STI/STD	0.321	0.028	1.477**	-0.096	-0.055	0.134
	(0.321)	(0.055)	(0.564)	(0.086)	(0.402)	(0.076)
**Likelihood of STI in next 6 months**–Reference: Little chance						
Some chance	-1.032**	-0.082	-1.356	-0.126	-1.305**	-0.101
	(0.337)	(0.056)	(0.701)	(0.095)	(0.432)	(0.094)
More than 50%	-1.665**	-0.092	-3.892***	-0.097	-1.044	-0.150
	(0.620)	(0.070)	(0.845)	(0.091)	(0.825)	(0.161)
Used other contraception	1.930***	-0.217*	3.239***	-0.017	1.689***	-0.321*
	(0.308)	(0.092)	(0.716)	(0.158)	(0.368)	(0.151)
**Condom lessons–**Reference: none						
Informal lessons	-0.209	-0.058	-1.092*	-0.040	0.407	-0.167
	(0.294)	(0.079)	(0.470)	(0.112)	(0.440)	(0.115)
Formal lessons	0.259	-0.071	-0.853	-0.021	0.949*	-0.226*
	(0.310)	(0.077)	(0.513)	(0.117)	(0.430)	(0.112)
**Region**–Reference: Atlantic provinces						
BC	-0.155	-0.030	0.504	-0.359	-0.157	0.242
	(0.457)	(0.124)	(0.831)	(0.192)	(0.638)	(0.166)
Prairies	0.133	0.030	0.792	-0.059	0.068	0.135
	(0.372)	(0.105)	(0.667)	(0.142)	(0.493)	(0.169)
Ontario	-0.174	0.076	0.423	-0.027	-0.291	0.179
	(0.340)	(0.097)	(0.596)	(0.121)	(0.460)	(0.161)
Quebec	0.901	0.123	3.674*	0.206	-0.420	-0.018
	(0.682)	(0.128)	(1.597)	(0.150)	(0.777)	(0.198)
Urban	-0.476	-0.059	-1.067	-0.124	-0.541	-0.030
	(0.292)	(0.069)	(0.561)	(0.099)	(0.409)	(0.103)
**Household income**–Reference: under $20,000						
$20,001-$40,000	0.202	-0.032	1.634	-0.060	-0.227	0.089
	(0.485)	(0.099)	(1.042)	(0.131)	(0.655)	(0.160)
$40,001-$60,000	0.006	0.044	2.071	0.072	-0.770	-0.004
	(0.477)	(0.094)	(1.115)	(0.128)	(0.624)	(0.154)
$60,001-$80,000	0.418	0.053	2.581*	0.025	-0.394	0.101
	(0.474)	(0.092)	(1.129)	(0.119)	(0.620)	(0.172)
$80,001-$100,000	-0.234	-0.072	1.000	-0.215	-1.124	0.060
	(0.492)	(0.100)	(1.135)	(0.132)	(0.654)	(0.173)
Over $100,001	0.549	0.159	2.014	0.072	0.326	0.164
	(0.510)	(0.093)	(1.167)	(0.121)	(0.703)	(0.189)
**Religion**–Reference: no religion or agnostic						
Catholic	0.349	-0.074	0.306	-0.032	0.453	-0.053
	(0.327)	(0.083)	(0.586)	(0.125)	(0.448)	(0.139)
Protestant incl. evan.	0.309	-0.077	1.248	-0.158	-0.395	0.170
	(0.388)	(0.089)	(0.726)	(0.129)	(0.555)	(0.144)
Other & PNA	-0.500	-0.114	-0.025	-0.265*	-0.777*	0.014
	(0.306)	(0.069)	(0.688)	(0.110)	(0.395)	(0.100)
How often do you attend religious services	-0.201*	0.019	-0.305*	0.016	-0.116	0.016
	(0.080)	(0.015)	(0.154)	(0.023)	(0.112)	(0.024)
Constant	1.538	1.990***	-0.530	2.007***	2.051	1.987***
	(1.096)	(0.274)	(2.889)	(0.281)	(1.338)	(0.421)
Observations	918		437		464	
N = zero	532		228		300	
F	1.772***		2.249		2.032	
AIC	2855.186		1486.030		1311.203	
BIC	3260.251	df = 84	1804.265	df = 78	1634.114	df = 78

Unstandardized multivariate regression coefficients with standard errors in parentheses. **Bold coefficients and standard errors** for sets of dummies for categorical variables indicate statistically significant tests for whether all group dummies are jointly equal to zero (the null hypothesis) using Stata 16 postestimation contrast command (p < 0.05).

*** p<0.001.

** p<0.01.

* p<0.05.

### Demographics

Consistent with previous studies, we find that several demographic factors influence patterns of condom use. As markers of social location, demographic factors shape social constraints, cultural and subcultural group membership, and social expectations within sexual encounters. Overall, our results indicate that age, education, relationship status, expectation of getting an STI in the next 6 months, use of another type of contraception, religious affiliation, and attending religious services are all consistently (including across cisgender subsamples) and significantly related to the probability that condoms were never used during the last 10 encounters, while controlling for other factors. Meanwhile, household income, relationship status, and gender and sexual orientation are significantly and consistently associated with the number of times condoms were used among those who used condoms. We find no significant association between condom use and self-reported sexual health, region of the country, urban vs. rural residence. Otherwise, results vary by sample or subsample.

Gender and sexual orientation influence patterns of condom use. Our primary finding on gender and sexual orientation is that gay, bisexual, or pansexual cisgender men use condoms during penile-vaginal intercourse significantly more cisgender women and heterosexual cisgender men do. As this sample is limited to participants’ reports of the last ten penile-vaginal sexual experiences, our results include a number of respondents with a lesbian, gay, or bisexual identity, who are reporting only on their last ten penile-vaginal experiences exclusive of any same-sex sexual interactions.

For dichotomous and continuous indicators, significance is determined by t-tests of the regression coefficients. This is denoted in Table 2 with *, **, or ***. For sets of dummy indicators for categorical variables, significance is based on a joint test of the null hypothesis that all coefficients for each category equal zero. In Table 2, when the probability that the null hypothesis for this joint significance test is less than 0.05, the group of coefficients and standard errors are **bold**.

**Fig 6:**
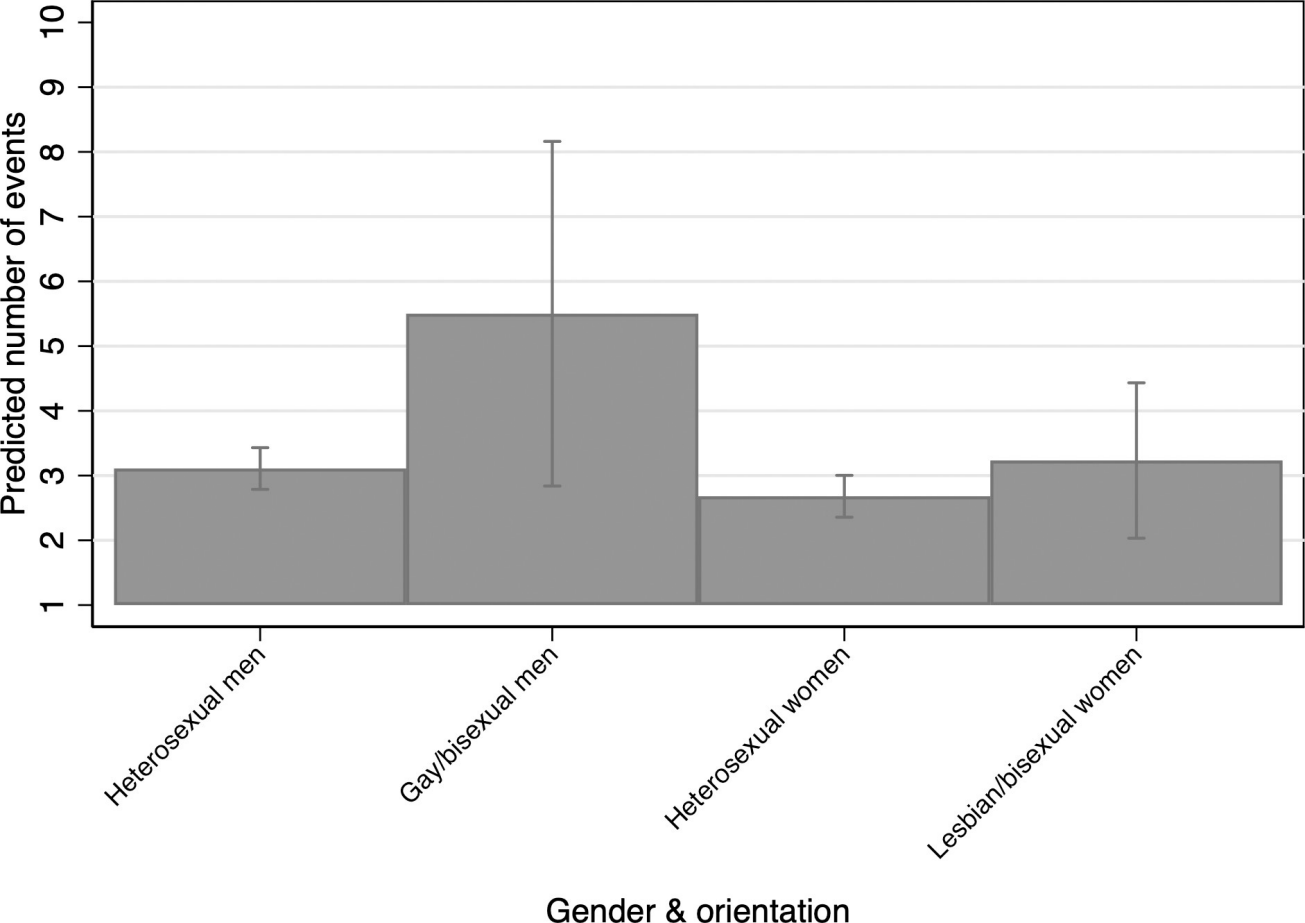
Predicted count by gender and sexual identity categories.

**Fig 7:**
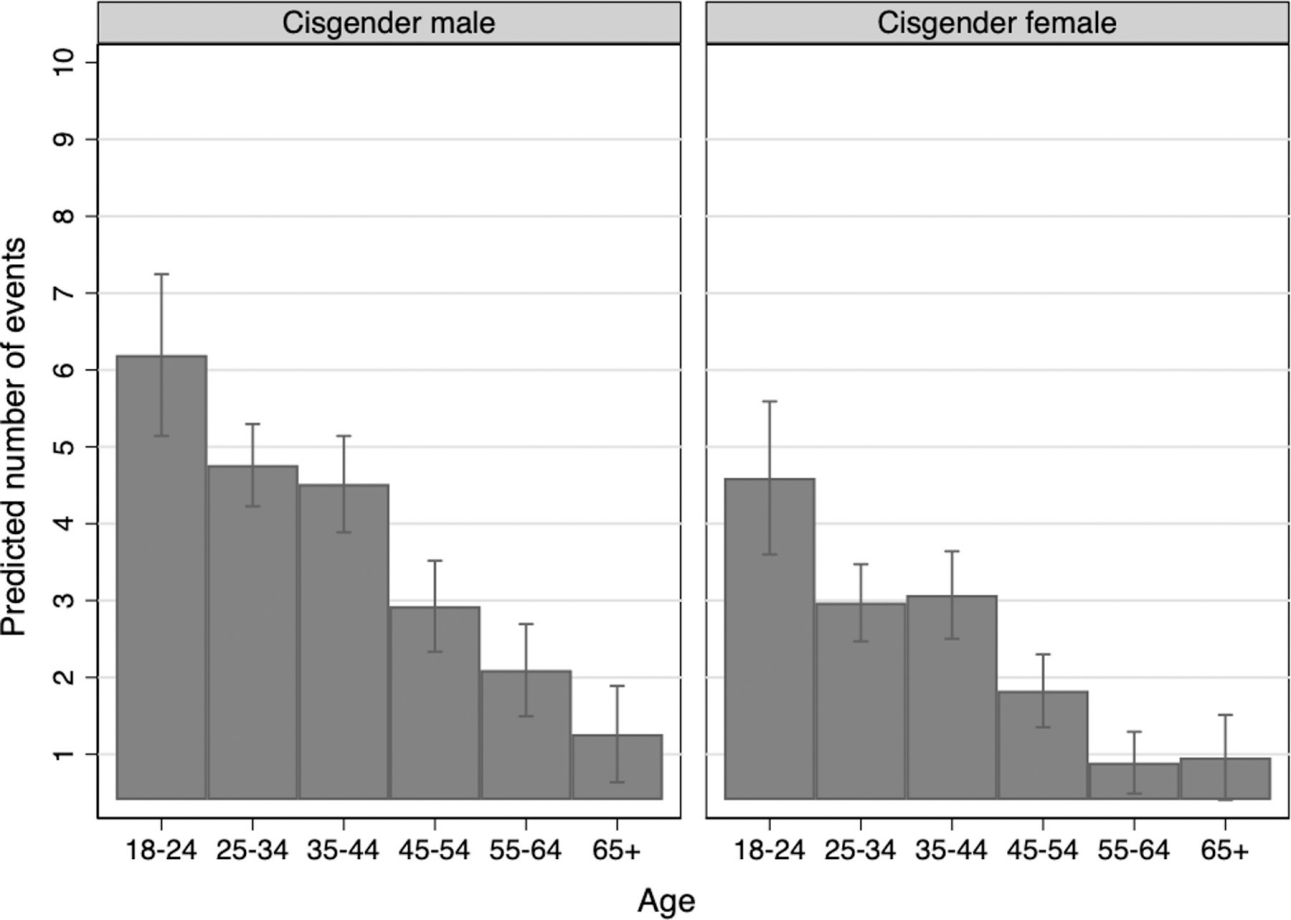
Predicted count by gender and age.

**Fig 8:**
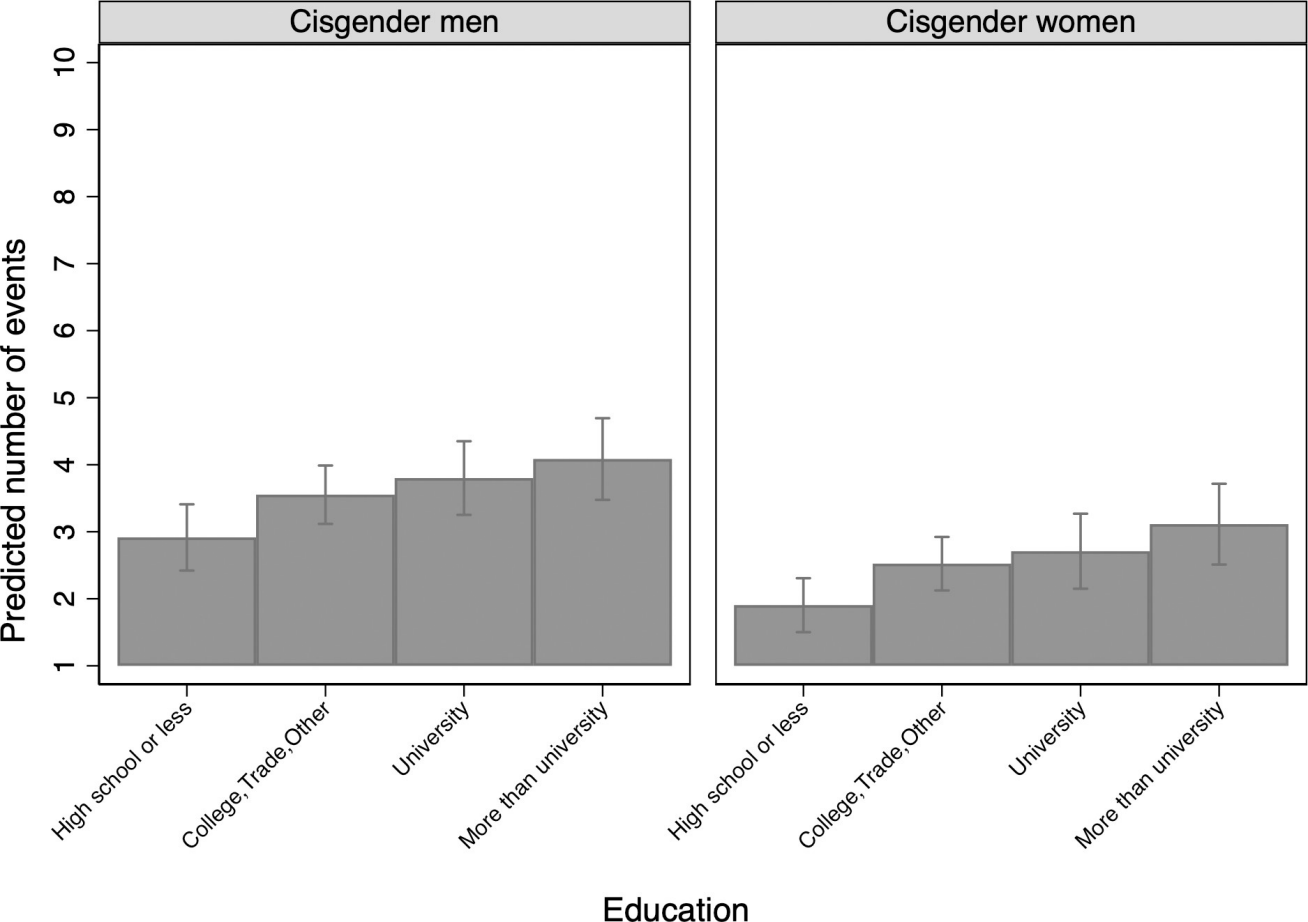
Predicted count by gender and education.

**Fig 9:**
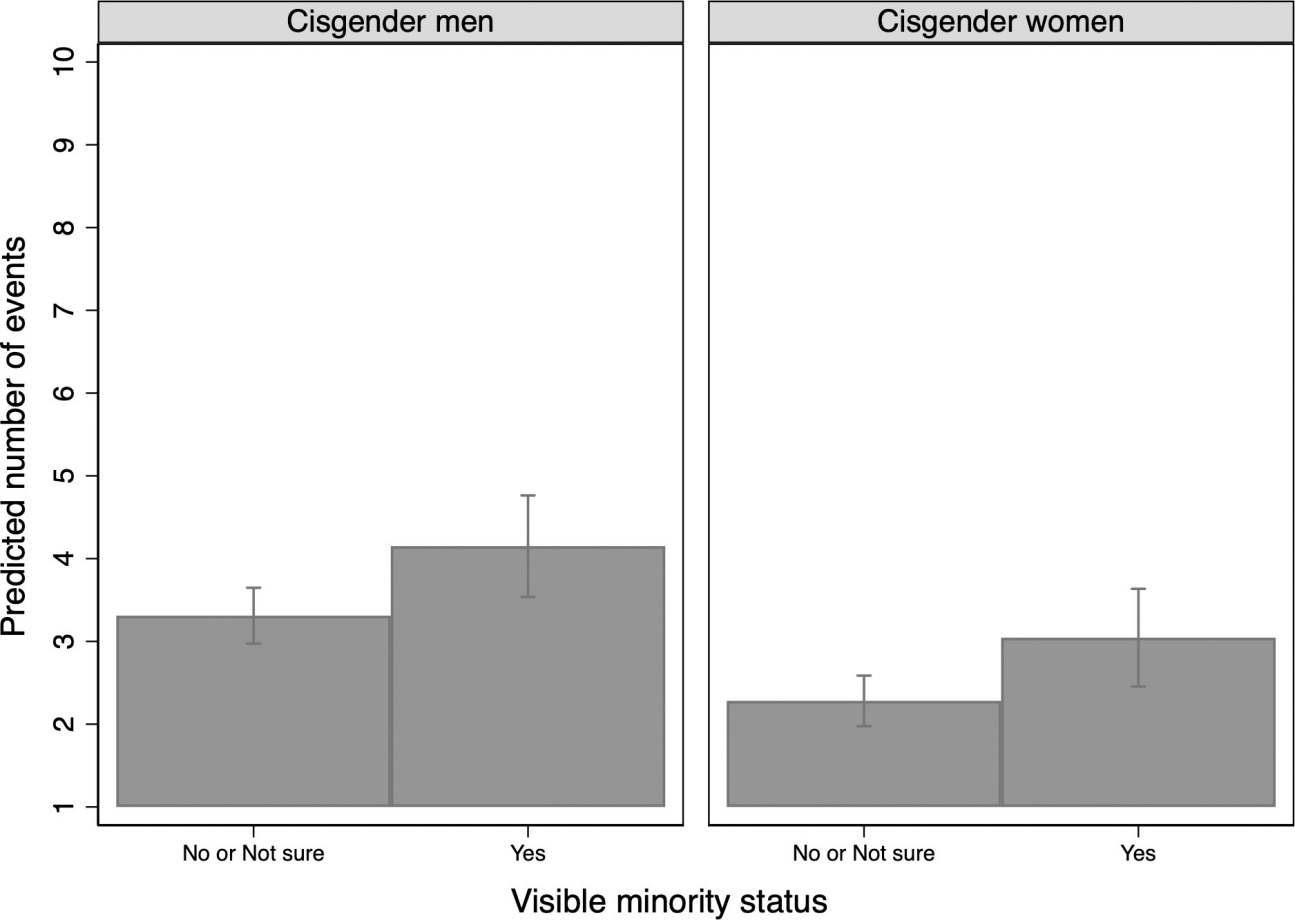
Predicted count by gender and visible minority status.

Using predicted probabilities holding all variables at their means, we examine condom use by intersecting gender and sexual identity categories. In Figs 6–9, the predicted probabilities are calculated based on the observed data using Stata 16’s margins command to estimate predicted probabilities over the categories of interest. In terms of these categories, gay/bisexual cisgender men have the highest predicted count of condom use in penile-vaginal sexual intercourse, and straight cisgender women have the lowest (Fig 6). While it is not appropriate to generalize these findings to gay men, lesbian women, or bisexual people as a group, it is instructive to see that sexual identity is an important predictor of condom use even for penile-vaginal intercourse only.

We find that age is negatively associated with condom use. Younger cohorts of adults use more condoms than older cohorts, even while controlling for other demographic characteristics. Older age groups are increasingly likely to report never using condoms, relative to younger groups. Predicted probabilities show that each ascending age group is less likely to use condoms (Fig 7). This finding holds for cisgender men and cisgender women.

Those with higher levels of education are more likely to use condoms than those with less education, though among those using condoms at all there is no significant difference in the frequency of condom use. In particular, those with more than a university degree are the least likely to never use a condom. Predicted frequency of condom use modestly increases with education level across cisgender subsamples, with cisgender men reporting higher overall frequency of condom use compared to cisgender women (Fig 8).

We find a positive association between visible minority group membership and condom use overall. Our models show that participants with self-identified visible minority status are significantly more likely than others to use condoms ever, though among those that used condoms at least once, condoms are not used significantly more frequently. The average predicted frequency of condom use across cisgender groups is higher for visible minority cisgender men than women (Fig 9).

We find some differences in the condom use patterns of Francophone and Anglophone Canadians. Among cisgender men, participants who expressed a preference to complete the survey in English have more frequent condom use than those who preferred French. We find no differences between Anglophone and Francophone women.

We find that relationship status matters to condom use patterns. Single people, whether not dating or dating, are both more likely to use condoms at all and more frequently than people who are married or living together in a relationship. These results are consistent when singles are dating someone regularly or have multiple sexual partners. Married participants and those living together as if married are more likely to report never using a condom in the last ten sexual encounters, and when they do use condoms, they do so less often than single participants.

### Sexual health

Our models include three variables related to sexual health, each of which is related to condom use. The first is a self-assessment of sexual health. While sexual health does not overall significantly explain condom used based on joint tests of statistical significance, the probability of ever using a condom, particularly among cisgender men, varies significantly according to self-reported sexual health. In particular, cisgender men who consider themselves in better sexual health are more likely to use condoms than those who consider themselves to have poor sexual health. Differences among women are not statistically significant, however.

Next, we include experience with a diagnosis of sexually transmitted infection (STI). Among cisgender men, having a medical history that includes an STI diagnosis is significantly associated with never using a condom in our analysis, while a prior STI diagnosis is not associated with the frequency of use among those that have used condoms recently. We find no association between STI diagnosis and condom use among women.

The expectation that contracting a sexually transmitted infection in the next six months is likely has a significant positive impact on whether participants ever used a condom. For cisgender men, the highest probability of ever using a condom is among men who think they have a greater than 50% chance of contracting an STI in the next six months. In comparison, cisgender women are significantly more likely to ever use a condom if they think they have even “some chance” of contracting an STI.

### Other contraception and condom education

The use of other forms of contraception is negatively associated with condom use, both the likelihood of ever using a condom and the frequency of using a condom among those that do. Among men and women, those who use other forms of contraception are less likely to use condoms than those without other forms of contraception. The association between types of previous condom lessons and condom use are not consistently significant among men and women. Cisgender men that report informal or formal lessons are significantly less likely to never use a condom than cisgender men who have had no lessons. Meanwhile, the associations between lessons and condom use among cisgender women are mixed. Only cisgender women with formal lessons report less frequent condom use than their peers with no lessons at all.

## Discussion

The current study provides valuable insights on the social organization of condom use in Canada. Using a representative sample of the Canadian adult population, this analysis speaks to condom use in a wide range of ages from early adulthood to advanced age, for diverse relationship statuses, among visible minority, among Anglophone and Francophone populations, and for differing educational levels. Our findings are limited to penile-vaginal intercourse in the past six months. Therefore, the reader should keep in mind that these findings exclude same-sex sexual encounters. Similarly, these findings are limited to analysis of the sexual encounters of participants who reported ten or more instances of penile-vaginal intercourse in the past six months. Participants with fewer sexual encounters were not analyzed here.

Similar to nationally representative samples in the United States and studies of particular age groups in Canada, this research finds that heterosexual, cisgender men use condoms more frequently than do heterosexual, cisgender women [8,12–14]. Younger people are the most likely to use condoms. Our findings also reflect past research confirming that single people use condoms more often than those who are married or living together [8]. We find that those who identified as visible minorities are significantly more likely to use condoms than others. Specifically, visible minority men used condoms more often than white men. This finding is consistent with findings in the United States [12], and it suggests a level of awareness of disproportionate risk of STIs within racially marginalized populations and an accompanying attention to condom use.

These findings, which confirm previous results elsewhere, make clear that condom use is not simply a matter of individual choice, but is a social act. One’s social location in terms of gender, sexual orientation, marital status and visible minority status are important influences on condom use behaviours. This body of evidence suggests that the social risks of using or not using a condom in a given sexual encounter are not evenly distributed in the population, and it recommends public health intervention strategies that take socio-demographic factors into account.

We examined three variables relating specifically to sexual health. First, cisgender men who self-assess as being in better sexual health are more likely to use condoms than those who think they are in poor sexual health. Second, men who have been diagnosed with an STI are significantly less likely to use condoms. And finally, perceiving that one will contract an STI in the next six months increases condom use. These results, along with our finding that condom use decreases as people age—in fact, older age groups are increasingly likely to report never using condoms—presents troubling confirmation of previous research that indicates increasing behavioral risk for STIs among large segments of the Canadian population who may feel that they are either not at risk or who practice risky behaviour even after contracting an STI [8,23]. These findings may be particularly troubling for public health advocates, who may hope that receiving a diagnosis of a sexually transmitted infection might increase future condom use. However, our results indicate that this is not the case, suggesting instead that those who avoid condom use do not change their behaviour even after receiving an STI diagnosis. The fact that perception of being at risk for an STI increases condom use supports public health agencies’ attention to awareness of STI risk in the promotion of condom use.

Our findings also point to the importance of education for increasing condom use. We find that men and women with higher education are more likely to use condoms. In general, the use of other forms of contraception negatively impacts condom use among those of all educational levels. This finding, consistent with previous research, is an important reminder to public health advocates that pregnancy prevention is an important motivator for condom use, although other forms of contraception offer insufficient protection against most sexually transmitted infections. The fact that many Canadians view their risk of acquiring an STI as very low may present a disincentive for condom use when another contraceptive is being used. Importantly, we find that instruction on how to use condoms significantly increases their use, whether that instruction comes as part of a formal sex education curriculum or from more informal social networks. These findings underscore the importance not only of educating the population on the risks for acquiring STIs that condoms are best at mitigating, but also on the need to provide the public with general information on condom use to enhance sexual experience. Further, among cisgender women, only formal rather than informal lessons are associated with increased condom use. This finding is important for sexuality education advocates and public health experts, who should pay particular attention to the gendered aspects of sexuality education impacts.

This study has several limitations. This research relies on self-reports of condom use among participants who were willing to complete a questionnaire about their sexual practices, attitudes, and relationships. Still, self-reports are not as reliable as daily diary studies. In addition, as a cross-sectional survey, our sample does not allow for analyzing trends of condom use over time. As this study focuses specifically on penile-vaginal sexual encounters, we cannot address condom use in same-sex sexual encounters, which are an important component of condom use in Canada. Because our respondents were limited to people who had engaged in penile-vaginal intercourse ten times in the past six months, our findings cannot be extended to those who have had fewer instances of intercourse. Finally, further research is needed to understand the contextual factors within particular sexual encounters that may encourage or discourage the choice to use a condom. As an analysis of the demographic, sexual health, and condom lesson predictors of condom use, this study cannot address the specific negotiations between sexual partners that produce decisions whether to use condoms.

Overall, this study provides a social analysis of condom use among adult Canadians. Our findings are consistent with other work in this field, demonstrating clearly that demographic, health, and educational factors are important inputs to the decision to use condoms. Our study supports the larger body of work that reveals social patterns in condom use. This is important information for public health efforts that seek to reduce the risk of STI transmission in the current era of increasing rates of sexually transmitted infections among adult Canadians.

In particular, it is vitally important that research continues to measure condom use patterns with repeated studies over time. As condoms continue to be a major frontline intervention in the transmission of sexually transmitted infections, updated knowledge of condom usage patterns is of vital importance both to researchers of sexual behaviour and to public health advocates. We argue that such research should continue to focus on the social patterns associated with condom use, the relationships between sexual health and condom use, and the effectiveness of educational practices on increasing condom use. As our study finds, these are significant predictors of condom use, showing that social context, health and education are all foundational to individuals’ choices to use condoms. An approach that focuses on only one of these aspects would be missing important components of condom usage patterns.
